# Close-to-Nature Silviculture to Maintain a Relict Population of White Oak on Etna Volcano (Sicily, Italy): Preliminary Results of a Peculiar Case Study

**DOI:** 10.3390/plants12102053

**Published:** 2023-05-22

**Authors:** Sebastiano Sferlazza, Guglielmo Londi, Donato Salvatore La Mela Veca, Federico Guglielmo Maetzke, Salvatore Vinciguerra, Giovanni Spampinato

**Affiliations:** 1 Department of Agricultural, Food and Forest Sciences, University of Palermo, 90128 Palermo, Italy; 2 Consultant Ornithologist, 51035 Lamporecchio, Italy; 3 Consultant Forester, 95031 Adrano, Italy; 4 Department of Agriculture, Mediterranean University of Reggio Calabria, 89122 Reggio Calabria, Italy

**Keywords:** species diversity indices, taxonomic distinctness, BACI study, old-growth forest, Calabrian black pine forests, vascular plants, birds

## Abstract

Habitat loss is a potential long-term effect of projected climate change for Mediterranean forest ecosystems. Here, we investigated the effectiveness of a close-to-nature silvicultural practice to conserve an old-growth white oak forest patch in Sicily (Italy) and promote regeneration dynamics. The study area, although small, is distinctive for its isolation, position and environmental characteristics. We conducted a Before–After Control–Impact (BACI) study to analyse the responses of different taxonomic groups (vascular plants and birds) to silvicultural treatments (selection thinning, no thinning), and to determine whether close-to-nature silviculture practices may cause significant shifts in the investigated communities. Specifically, we assessed the responses of (1) vascular plants by means of species diversity and taxonomic distinctness indices and (2) birds in terms of diversity, abundance and forest specialisation. Preliminary results suggest that cautious close-to-nature silviculture practice could—by mimicking natural gap dynamics—contribute to maintaining old-growth forest patches and promote oak seedling emergence without short-term detrimental impacts on biodiversity. Although the monitoring has to be repeated over the long-term, the multi-taxon approach and indices incorporating information on taxonomic relationships into diversity measures were demonstrated to be valuable tools for interpreting biotic community structure and dynamics.

## 1. Introduction

Mount Etna (3324 m above sea level), on the eastern coast of Sicily (Italy), is one of the most active volcanoes in the world, and the highest in continental Europe. Etnean forests have been shaped in their extent, composition and structure by more than two millennia of interactions among geological history, climate, natural disturbances and anthropogenic pressures. The almost continuous eruptive activity and anthropogenic activities (forest harvesting, wildfires, grazing, land-use changes) have been key drivers influencing forest dynamics [1] and habitat fragmentation [2] on the Etnean territory.

The importance of biodiversity conservation has led to the establishment of a network of protected areas (a regional park and some Nature Reserves, in addition to Natura 2000 sites) with different levels of protection in the Etnean territory. Protected areas now play a crucial role in proactive adaptive management for tackling climate change [3,4,5], posing new challenges for managers and decision makers of the 21st century [6]. The effects of impact caused by climate change affect forest resilience by altering the frequency and intensity of drought, wildfires, heat waves, insect and pathogen outbreaks, flash floods, wind and snowstorms. In Mediterranean ecosystems, habitat loss is one of the long-term consequences of predicted climate change.

Despite the long history of natural and anthropogenic pressures, it is still possible to find patches of old-growth forests in small and remote areas of Mount Etna. Old-growth forests are late-successional forests with high levels of structural complexity compared with early-successional forests [7,8]. They play an important role in biodiversity conservation [9] and offer a wide spectrum of ecosystem services, including carbon and water flux regulation [10,11,12,13]; moreover, they represent a reference point for improving current sustainable forest management practices [14]. Thus, the use of management practices emulating natural disturbances and dynamics has been explored in several studies aimed at maintaining, promoting and restoring old-growth attributes at the stand scale [15,16] in order to improve carbon storage [17], soil physical traits [18], tree growth [19] and the biological diversity of different taxonomic groups, such as invertebrates [20], vascular plants [21,22,23] and birds [24,25,26,27].

In this study, we focused on a relict population patch of white oak (*Quercus congesta* C. Presl) close to old-growth conditions within an even-aged Calabrian black pine (*Pinus nigra* subsp. *calabrica* (Delam. ex Loudon) A.E. Murray) plantation in a protected area of Etna Regional Nature Park. Pines were planted in the late 1970s as preparatory species for fostering the maintenance and regeneration of the old oak trees. The pines were not tended thereafter, resulting through time in forest canopy closure. Therefore, the forest currently consists of a two-layered stand: the upper layer is dominated by Calabrian black pines, while old white oaks occur as single isolated individuals or in small patches in the bottom layer. The interspecific competition between the tree species tends to be high, producing an all-sided suppressive effect of the top-layer pines on the lower-layer oaks. Considering that gaps in the forest canopy can play an important role in affecting the structure of forest stands and plant succession [15,28], as well as soil microclimate and nutrient cycling [17,29], in this work, the preliminary effects of a prudent thinning intervention on the pine layer carried out in 2015 were evaluated through various indicators, with respect to a control (unthinned) part of the stand.

The dispersal performance of white oak and the response of seedlings, in terms of establishment and growth, in addition to changes in understorey diversity [22,30] and cover [25,31,32,33,34] were studied; moreover, the potential distribution and composition of the ornithological community were also taken into account, as these are considered good indicators of biodiversity [35,36]. The main aim was to evaluate the possible recovery of old-growth forest patches over a long time scale and the effectiveness of regeneration of white oak, which is the basic prerequisite to maintain the local population and preserve its genetic variability.

## 2. Materials and Methods

### 2.1. Study Area

**Figure 1:**
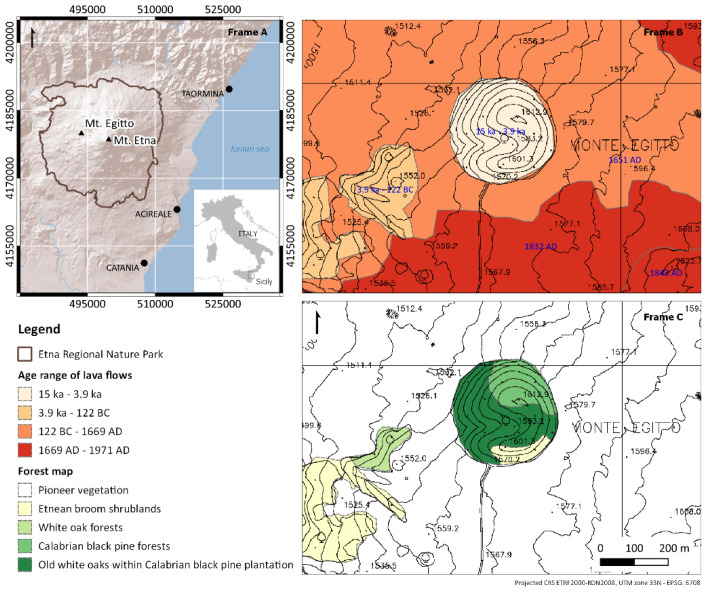
(Frame **A**) Location of Mount Egitto on the western flank of Mount Etna volcano in Sicily (Italy), with the Etna Regional Nature Park superimposed on the shaded relief in brown; (Frame **B**) Historical lava flows and their ages (blue label) for Mount Egitto and its surroundings according to a geological map of Etna volcano (adapted from Branca et al. [38]); (Frame **C**) Forest map of the study area and its surroundings.

The study was carried out on Mount Egitto, a forested tephra cone located on the western flank of Etna volcano (Lat. 37°45′57″ N, Long. 14°55′40″ E). The origin of this cone has been related to the flank eruptions that occurred between Ellittico caldera (about 15 ka) and Il Piano caldera (122 BC, Plinian eruption) [37]. Mount Egitto has a surface of 8 ha and is located at altitudes ranging from 1529 to 1612 m a.s.l., surrounded by poorly vegetated lava flows produced during the 1651, 1832 and 1843 AD eruptions [38] (Figure 1). Mount Egitto is subject to a temperate oceanic bioclimate, supra-Mediterranean thermotype and hyperhumid ombrotype [39]. The soils are classified as Leptosols [40]. The forest is dominated by Calabrian black pine (*Pinus nigra* subsp. *Calabrica*) of both natural and artificial origin. The local population of black pine is referred to as a subspecies endemic to southern Italy, with a natural range extending from Calabria (Sila and Aspromonte massifs) to Sicily (Etna volcano). Calabrian black pine forests are a priority habitat (cod 9530* (Sub-)Mediterranean pine forests with endemic black pines) according to the ‘Habitats’ 92/43/EEC Directive [41]. Old white oak trees grow as single isolated individuals or form small patches within the Calabrian black pine plantation matrix. *Quercus congesta* is an acidophilous and orophilous deciduous oak and is endemic to Sicily, Calabria and Sardinia [42], belonging to the *Quercus pubescens* cycle, although recent studies have questioned the taxonomic autonomy of this species [43,44,45]. White oak forests are also a priority habitat (cod. 91AA* Eastern white oak woods) according to the above-mentioned Directive. This species is recorded as ‘least concern’ in the International Union for Conservation of Nature’s (IUCN) Red List of Threatened Species [46]. Historically, white oak forests covered vast areas in south and southeastern Europe, but their extent has undergone intense shrinkage and fragmentation due to anthropogenic and natural disturbances, such as wildfires, grazing, logging and land-use changes [47]. Today, *Quercus congesta* grows in mixed acidophilic stands in fragmented areas on the main mountain ranges of Sicily [48]. Among the numerous vascular plants occurring in the study area, *Genista aetnensis* (Raf. ex Biv.) DC.—a giant broom endemic to Corsica, Sardinia and Mount Etna—is one of the most widespread, and covers the entire southern sector of the cone. Finally, it is worth noting that Mount Egitto represents the only known Italian locality for the mushroom *Sporidesmium bacidiicola* Alstrup and the lichen *Waynea giraltiae* van den Boom [49]. Maetzke et al. [50,51] provided the following preliminary characterisation of the structural attributes of the investigated old-growth forest patch: (1) 70 old oaks with diameter at breast height (DBH) greater than 40 cm, (2) a total amount of deadwood of 30 m^3^ ha^−1^, (3) more than 60% of deadwood occurring in the latest decay classes. The occurrence of old-growth features has been linked to the combined effect of local factors, such as climate, altitude, soil, periodic volcanic ash falls and lava flows, distance to rural areas and low levels of anthropogenic pressure [50].

### 2.2. Survey Design

We carried out a Before–After Control–Impact (BACI) study to assess changes in the vascular plant and bird communities in response to close-to-nature silviculture practice. The monitoring activity started in May 2015 (before treatment) and ended in May 2018 (3 years after treatment). In such surveys, a BACI study is appropriate because it allows for referring changes in community diversity to an impact, rather than to natural variability [52]. Concerning the treatment, a selection thinning (T)—namely, a close-to-nature silviculture practice that favours the old white oaks (target trees) by removing their crown competitors (Calabrian black pines)—was applied in late summer/early autumn 2015. The control—i.e., no thinning (C)—sites received no intervention/practice, which represents the current management approach in the regional park.

We adopted the principle of ‘free choice of felling’ [53]: the thinning was carried out tree-by-tree on a case-by-case basis, aimed at creating a gap of 500–2000 m^2^ in relation to the size of oaks trees, with the goal to rid the oaks’ canopy of intersecting pine trees. A total of 10 gaps of different sizes were created, including 4 gaps of 500 m^2^, 3 of 1000 m^2^, 2 of 1500 m^2^ and 1 of 2000 m^2^. The low number of gaps was used for two reasons: firstly, the focus was only the relict population of white oak occurring as single isolated individuals or as trees grouped in small patches, and secondly, removal of canopy trees in a protected area is highly regulated by the Etna Regional Nature Park’s policy.

Based on a preliminary field survey, we first defined the thinning sites and nearby control sites (generally within a 50–100 m distance) that were the same oak–pine habitat type and had similar canopy and understorey cover patterns. The sample size was established by applying a sampling design proportional to treatment sites and forest size. Vascular plants were sampled along 11 linear, georeferenced permanent transects according to the methodological protocol of Annex I Habitat monitoring [54]. The taxonomic nomenclature followed Pignatti [55,56,57]. We conducted an inventory in May 2015 and in May 2018, reducing any possible temporal bias in data collection. Each transect consisted of adjacent 1 m^2^ (1 m × 1 m) plots in a repeating pattern; the total transect length varied from 26 to 52 m and was determined according to old-growth oak density, with the aim of including 1–5 old oaks per transect. In total, 7 and 4 transects were established in the thinning (~5 ha) and control (~3 ha) sites, respectively. All vascular plants (including trees, shrubs and herbaceous species) <2 m in height occurring within the transects were recorded. We made a distinction between seedlings (young plants raised from seed not yet 1 m high), saplings (young trees about 1–2 m high and 2–4 cm in dbh) and tree individuals (dbh ≥ 4 cm) [58]. Canopy coverage of each vascular plant species was also estimated by assigning one of the following twelve coverage ranges: 0–1%, 1–5%, 5–10%, 10–20%, 20–30%, 30–40%, 40–50%, 50–60%, 60–70%, 70–80%, 80–90% and 90–100%. The species observed along the transect were distinguished into nemoral and non-nemoral in accordance with their membership in the phytosociological classes *Querco roboris–Fagetea sylvaticae* Br.-Bl. & Vlieger in Vlieger 1937 or *Quercetea ilicis* Br.-Bl. in Br.-Bl., Roussine & Nègre 1952 [59,60,61].

Birds were surveyed using a point counts method [62]. Censuses were conducted during the breeding season in May 2015 and May 2018. We selected 21-point counts spaced over 50 m apart, non-randomly located across the whole study area (8 ha). Each of the 21 sampling points was surveyed 3 times in each study year. Within this period, sampling was replicated at intervals of 8 days. Each point count was characterised by the following variables: applied management (10 points were in thinning sites—with at least 25% of the area within a 25 m radius of the point having been thinned—and 11 points were in control sites), vegetation structure and composition (pine plantation coverage and number of old oaks within a 25 m radius of the point). All birds seen and/or heard were recorded over a 5 min period, and their distances were noted according to 3 bands (0–25 m, 25–50 m, >50 m). Point counts were conducted within 5 h of sunrise and in a random order across the study area, reducing any possible temporal bias in data collection. Observations of over-flying birds, i.e., those that did not land in trees or on the ground, were excluded. Additionally, migrant birds that were apparently flying high over the study area without stopping to use it stably were not counted. For the analyses, given the small size of the study area and the proximity of sampling points, we only considered birds detected within the first distance band (0–25 m) to avoid double counting among contiguous points.

### 2.3. Data Processing and Analysis

#### 2.3.1. Vascular Plants

We used species and taxonomic diversity indices to analyse the short-term effects of silvicultural treatment on the vascular plant community. Five species diversity indices were calculated for each survey time (before and after treatment) at each treatment (thinning and control): the species richness, Shannon–Wiener, Pielou’s evenness, complement of the Simpson and nemoral species indices. Species richness (*S*) was computed by counting the number of species occurring in each sample; it is often used as a biodiversity indicator for assessing management effects [63,64]. Shannon–Wiener’s index (*H′*) [65] is one of the best known and most widely used of all diversity indices [66], combining species richness (the number of species present) and species abundance (the number of individuals per species). It was calculated as Equation (1):

(1) $$H^{\prime }=-\Sigma p_{i}ln(p_{i})$$

To assess the homogeneity of each sample, Pielou’s evenness index (*J′*) [67] was computed as Equation (2):

(2) $$J^{\prime }=\frac{H^{\prime }}{ln(S)}$$

where *S* is the total number of species recorded in each sample. The complement of the Simpson index (1 − *D*) [68] estimates the probability that two randomly chosen individuals from a sample belong to different species. It was computed as Equation (3):

(3) $$1-D=1-\Sigma p_{i}^{2}$$

For both *H′* and 1 − *D* indices, *p_i_* is the relative coverage of the *i*th species (*p_i_ = n_i_/N*), *n_i_* is the coverage of the *i*th species, and *N* is the total coverage. The nemoral species index (*N*) was computed as Equation (4):

(4) $$N=\Sigma p_{i}$$

where *p_i_* was calculated as the ratio between the coverage of the *i*th nemoral species (*n_i_*) and the total coverage (*N*). The occurrence of species strictly associated with a nemoral context on Mount Egitto may help us to understand the habitat structure and its dynamics. Conventional species diversity indices summarise the information about the relative abundances of species within a community or sample without regard to the differences between species or their degree of taxonomic relatedness [69,70]. If two communities have identical numbers of species and equivalent patterns of species abundance, but differ in terms of taxonomic diversity, it seems intuitively appropriate that the most taxonomically varied community is the more diverse [66]. Furthermore, the taxonomic diversity can reveal the effects of different forest management techniques on diversity that are not measurable by conventional indices that do not incorporate species relatedness [71]. The hierarchical Linnaean classification was used as a proxy for cladograms representing the relatedness of individual species to compute taxonomic diversity indices. For each survey time at each treatment, a composite taxonomy was compiled, and six taxonomic levels were considered (species, genus, family, order, sub-class and class). Two taxonomic diversity indices designed by Clark and Warwick [72,73] were computed from species abundances using coverage data:

(5) $$\Delta^{+}=\frac{\Sigma \Sigma_{i<j}\omega_{ij}}{s(s-1)/2}$$

(6) $$s\Delta^{+}=\underset{i}{\Sigma }\left[\frac{\Sigma_{i\neq j}\omega_{ij}}{\left(s-1\right)}\right]$$

where *ω_ij_* is the distinctness weight given to the path length linking species *i* and *j* in the taxonomy, and *s* is the species observed. Average taxonomic distinctness (Δ^+^, Equation (5)) is defined as the average taxonomic path length between any two randomly chosen species, and is independent of species richness [72,73]. Average taxonomic distinctness (Δ^+^) is multiplied by species richness (*S*) to give the total taxonomic distinctness (*s*Δ^+^, Equation (6)). It is a useful measure of the total taxonomic breadth of a community—as a modification of species richness—that allows for species inter-relatedness [72].

We then tested the effect of the treatment (thinning, control) and time (before and after) on five species diversity indices and five taxonomic diversity indices of the vascular plants. First, we performed a normality test, checking for the normality assumption for ten indices per dataset. An unpaired two-sample *t*-test was performed to test the effect of the treatment (thinning vs. control) at fixed survey times. The homogeneity of variances was first checked using a F-test. Pairwise comparisons were applied to test the effect of the time (before vs. after) per fixed treatment: we performed paired-sample *t*-tests and—when the datasets were non-normally distributed—the Wilcoxon signed-rank test. The relationships between the ten diversity indices were examined by correlation analysis based on Pearson’s product–moment correlation coefficient (*r*). The analyses were carried out using the software program R, version 3.6.3, packages: car, Hmisc, nortest, pastecs, psych and vegan [74]. For all statistical analyses, the significance level was at *p* < 0.05.

#### 2.3.2. Birds

The bird community was described by the species frequency and abundance of individuals at each survey time. A non-parametric Wilcoxon signed-rank test was used to test the effect of time (before vs. after) on total species richness and total abundances, as these data did not meet the assumptions of parametric statistics. We described the degree of forest specialisation of bird communities. Specifically, we narrowed down the full list of birds to forest specialists—that is, species linked to forest habitats, excluding species that also use other, non-forested habitats—thus calculating three indices: species richness, abundance and Woodiness Bird Community Index (WBCI). The last derives directly from scores that describe the response of common species to local wood-cover, at the Italian scale [75]. WBCI was calculated as the sum of the scores of all forest specialists in each point count: the higher the WBCI, the more specialised the forest bird community. We also used the scores of the species defined in Londi et al. [75] to objectively select forest specialists, that is, the species whose score exceeded 0.5. We performed paired-sample *t*-tests to test the effect of time on the forest specialisation community indices, as these data met the assumptions of parametric statistics. Finally, we performed general linear models (GLMs) [76] to test for effects of the environmental variables, namely, treatment, vegetation structure and composition (pine plantation coverage, number of old-growth oaks), on the bird community. We ran models for all possible combinations of the variables (including survey time) and selected the best model according to Akaike’s information criterion corrected for small samples (AICc); we also examined models where ΔAICc < 2 [77]. GLMs were calculated for the total species richness, total abundance and the three forest specialisation indices. We also calculated GLMs for a total of 16 individual bird species (abundance data for all those averaged more than for individuals per survey time, presence/absence data for those averaged 2–4 individuals per survey time). We used Gaussian distribution (link function = identity) in WBCI models, negative binomial distribution (link function = logit) in models for species with presence/absence data, and Poisson distribution (link function = log) in all other models [78]. All statistical analyses were performed with R version 3.6.3 software, using the package AICcmodavg [74]; the significance level was set at *p* < 0.05.

## 3. Results

### 3.1. Vascular Plants

During the study period, a total of 45 vascular plant taxa, belonging to 40 genera and 22 families, were recorded (Table S1 in the Supplementary Material). Poaceae, Fabaceae, Rosaceae and Asteraceae were the families with the largest number of species (10, 6, 5 and 3, respectively), together accounting for 53% of all recorded species; the remaining 18 families contained only 1 or 2 species. Before treatment, we recorded a total of 31 vascular plant species, belonging to 28 genera and 14 families. After treatment, the checklist included 40 vascular plant taxa, belonging to 37 genera and 20 families. Overall, we recorded 14 new occurrences of vascular plant taxa, accounting for 35% of the checklist after treatment (Table S1): 10 species were found in thinning sites (*Allium longispathum*, *Galanthus nivalis*, *Muscari commutatum*, *Crepis leontodontoides*, *Doronicum orientale*, *Linaria purpurea*, *Erysimum aetnense*, *Ranunculus neapolitanus*, *Fragaria vesca* subsp. *vesca*, *Vicia cracca*), 1 in controls (*Myosotis sylvatica* subsp. *sylvatica*) and 3 in both sites (*Anthoxanthum odoratum*, *Galium aparine* and *Vicia disperma*); in contrast, 5 species disappeared from the sites following treatment (*Festuca circummediterranea*, *Phleum hirsutum* subsp. *ambiguum*, *Monotropa hypopitys*, *Quercus ilex* and *Potentilla calabra*). Moreover, 43% of the new occurrences established during the period 2015–2018 were nemoral species strictly associated with the local species pool (Table S1).

After treatment, white oak seedlings increased their relative coverage by 22–335% in thinning sites and 35–120% in control sites, while white oak saplings increased their relative coverage by 25–155% in thinning sites and 44–330% in control sites.

**Figure 2:**
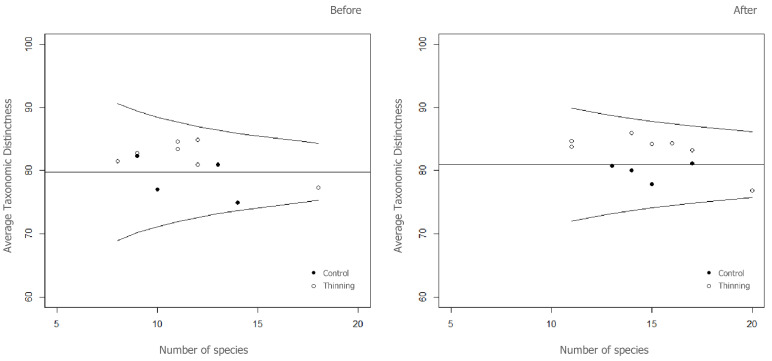
Funnel plots showing average taxonomic distinctness (Δ^+^) versus number of species before and after treatment for vascular plants. The 95% probability funnel is shown as curved lines, while the theoretical mean is shown as a horizontal line. Filled and empty points represent the control and treatment (Thinning), respectively.

None of the 7 diversity indices showed significant differences (*p* > 0.05) between the treatment (thinning) and the control per survey time. Results from the pairwise comparison indicated a significant effect of time (*p* < 0.05) following thinning treatment in only 3 indices: species richness (*S*), Pielou’s evenness (*J′*) and total taxonomic distinctness (*s*Δ^+^) (Table 1). After the treatment, *S* and *s*Δ^+^ were on average 28% and 30% higher in thinning and in control sites, respectively. *J′* decreased ca. 10%, while nemoral species (*N*) increased ca. 15% in both sites during the 3 years of monitoring. Average taxonomic distinctness slightly increased after treatment (Table 1), and Δ^+^ values were within the 95% confidence funnel (*p* > 0.05) in all sites (Figure 2), suggesting a good degree of taxonomic stability. Following treatment, values of Δ^+^ for all control sites fell below the theoretical mean, indicating that the control sites had lower average taxonomic range than most of the thinning sites and the entire study area.

The correlations among diversity indices computed for different datasets are shown in Tables S2–S4 in the Supplementary Material. No general patterns were detected when species diversity and taxonomic distinctness indices were correlated in each dataset.

### 3.2. Birds

Overall, 29 bird species were recorded (Table 2). A total of 25 species were recorded before treatment, 26 after treatment, and 22 were present at both survey times. For the 0–25 m band, a total of 21 species were detected (Table 2); 19 and 18 species were recorded before and after treatment, respectively, while 16 species were present at both survey times. *Upupa epops*, *Lullula arborea*, *Phoenicurus ochruros*, *Sylvia cantillans*, *Pica pica*, *Corvus cornix*, *Linaria cannabina* and *Emberiza cirlus* that occurred along the forest’s edge are typical of open or shrubby habitats. Other bird species were associated with wooded areas. Before treatment, *Periparus ater*, *Cyanistes caeruleus* and *Phylloscopus collybita* were the most common species, with frequencies of occurrence higher than 0.50; *Sylvia atricapilla* was also rather common, with a frequency of 0.48, followed by *Troglodytes troglodytes*, *Sitta europaea* and *Certhia brachydactyla*, with frequencies close to 0.30. After treatment, *C. caeruleus*, *P. collybita*, *P. ater* and *C. brachydactyla* were the most common species, with a frequency higher than 0.50, followed by *T. troglodytes*, *S. atricapilla*, *S. europaea* and *Fringilla coelebs*, with frequencies between 0.30 and 0.50. *P. ater* and *C. caeruleus* were the most abundant species in both survey times; *P. collybita* was also abundant across time, followed by *S. atricapilla*, *C. brachydactyla*, *S. europaea* and *T. troglodytes*. Selection thinning had a significant positive effect on *C. brachydactyla* and *F. coelebs*. The pine plantation had a significant positive effect on *Erithacus rubecula* and *S. atricapilla*, and a significant negative effect on *C. brachydactyla*, *F. coelebs* and *S. europaea*. Following treatment, we observed a marked increase (ca. four times) of *Garrulus glandarius* in terms of frequency and abundance (Table 2). The species richness and abundance of the bird community observed before the treatment were not significantly different compared to those observed after the treatment; furthermore, we found no significant effect of environmental variables on these community indices (Table 3). Finally, no significant effect of the time and environmental variables was detected on the forest specialisation community indices (Table 4). Our results show that the list of the most common and abundant species—as well as forest specialisation community indices—seemed to remain relatively unchanged despite the treatment, suggesting that the bird community remained stable over time.

## 4. Discussion

Given that the objective of the ongoing work is to evaluate the effectiveness of natural forestry in maintaining the relict population of white oak, the results will be analysed in relation to the preliminary effects that the first interventions have had, albeit in a short time. The close-to-nature forestry approach was chosen in the belief that the relict stand of old oak should be freed from competition in a very prudent and progressive way. Therefore, the first thinning intervention was very cautious and limited in space, with the awareness that we will have to proceed carefully also in future interventions. As discussed below, our considerations are still very limited, given the short period of observations. On the other hand, it was necessary to evaluate the situation before and shortly after thinning so as to have a basis for future decision making, using a trial-and-error method. For the purposes of the ongoing evaluation, we also checked which indicators were the most suitable to evaluate possible changes.

### 4.1. Vascular Plants

The results showed that the increased light availability resulting from thinning and soil surface disturbance by silvicultural operations could have a slightly positive effect on the emergence of both nemoral and non-nemoral seedlings in the treated areas. In particular, we observed an increase in the coverage of white oak seedlings after thinning that was more than double than in control plots. Considering these data, it seems that at least the initial phases of white oak recruitment are rather effective in gaps, as well as under a closed canopy, with a notable numerical increase of both seedlings and saplings, though the rate of the latter is significantly higher in the control. As a matter of fact, however, it must be distinguished between regeneration and effective recruitment. Tree regeneration can be decomposed into several subprocesses, starting with seed production, followed by seed dispersal, seed storage, germination, establishment of seedlings, growth of seedlings and saplings, and finally, the recruitment of small trees that exceed a certain measurement threshold (see, e.g., Price et al. [79]). In our study, despite the numerical abundance of saplings in control plots, no young tree was actually recorded, suggesting that the regeneration processes stop anyway at this stage. Most likely, due to unsuitable light conditions caused by the excessively closed canopy, saplings progressively decline to death, without ever managing to develop into adults. On the contrary, it is expected that thanks to the clearings created in the thinned plots, at least a rate of the established saplings can grow up and move to the successive development classes, therefore contributing to replace the senescent oak trees. According to that, in order to validate our assumptions and eventually adopt the necessary adjustments, it is of basic importance to go on with regular and continued monitoring in the next years.

Vascular plants showed a slight increase in species richness values only in thinned sites, probably due to a more heterogeneous environment—new species enter, others disappear and some slightly change their density. Higher plant species’ richness in managed rather than in unmanaged forests has been reported in several small-scale forestry studies from temperate and boreal regions [23,63,64,80]. Several factors—and combinations of them—may explain the positive impact of selection thinning on species richness. In fact, vascular plants might benefit from (1) higher availability and heterogeneity of resources (such as light and nutrients) because of canopy openings [63,64,81,82,83,84,85], (2) litter removal and soil disturbance [23,82,83], (3) seed bank activation following soil disturbance [86,87] and (4) lower amounts of pine litter inhibiting seed germination and seedling performance [88,89]. Three years after selection thinning, evenness was significantly lower; this result could be explained by the increase in plant species richness. White oak was the most common species to emerge after thinning. Furthermore, nemoral species showed slightly higher, though not significant, values in thinning sites. This result disagrees with the findings of some studies that indicated that specialist species are expected to be negatively influenced by small patch size compared to generalist species with a wider tolerance [90,91]. Mount Egitto is like a wooded island that has risen within recent lava fields, by far colonised by sparse light-demanding herbaceous and subshrub vegetation. The lack of forests in the surrounding areas can, therefore, explain the non-statistically-significant increase in nemoral species.

Although several studies have shown that average taxonomic distinctness is suitable to monitor the effects of natural and anthropogenic disturbances on biotic communities [70,73,92,93], in our study, only total taxonomic distinctness was able to discern significant changes in the vascular plant community at least three years after selection thinning. The slight but significant increase in species richness and total taxonomic distinctness may be mainly due to an increase in non-nemoral species from the adjacent areas towards the cone, given the non-significant increase in the nemoral species index. This evidence suggests that the increment or decrement in the number of species is one of the best disturbance indices and is, therefore, essential when differentiating an ecosystem’s ecological status [94]. These results can be considered a starting point to address forest management towards the maintenance or improvement of plant community biodiversity.

### 4.2. Birds

Despite its small forest patch size, Mount Egitto hosts a rich and heterogeneous bird community. Total bird species richness was higher compared to those recorded in similar forest habitats (e.g., mature oak wood, pine wood, pine wood mixed with broadleaved trees) on Mount Etna [95,96] and close to the highest richness values reported in the woods of the whole of Sicily [32]. The impact of the treatment on the bird community showed no significant variation based on the set of diversity indices used, although the total richness appeared to increase in species linked to pine cover and those typical of open habitats and forest edges. We also recorded an increase in frequency and abundance of some forest specialists in thinned sites, such as *C. brachydactyla* (associated with old trees and mature forest stands) and *F. coelebs*. On the other hand, while *G. glandarius* increased over time, there was no significant evidence for the role of thinning in this process. Our findings partially contrast with those obtained in other studies of bird responses to thinning in different regions [24,97,98,99]. We should also take into account that due to the small size of the study area, some changes could remain undetected in statistical analyses: *S. europaea*, a species associated with mature stands, showed a quasi-significant (*p* < 0.1) positive effect from selection thinning, and *Sylvia cantillans* seemed to have increased after thinning (the species was recorded in four points in 2018, vs. only two points in 2015), though analyses did not show significant effects. However, it is important to note that our forest patch is close to old-growth conditions with respect to previously cited studies, and that the oaks stands positively affect specialist species’ richness and abundance [100]. Moreover, large old trees play a fundamental role in driving small-scale environmental heterogeneity and generating the sets of characteristics suitable for cavity nesters or bark-feeding species [101]. As a result, invertebrates and arthropods could proliferate because of a higher abundance of suitable microhabitats, enhancing the food availability for various bird guilds. Our findings are consistent with previous studies [100,101,102,103] showing that forest heterogeneity in both age and structure has important positive effects on overall richness and specialist species’ richness. The lack of major significant differences resulting from silvicultural treatment in the computed indices—as well as for bird species themselves—suggests that selection thinning as practiced on our study area did not change the quality of the habitat for breeding bird populations or communities. However, the increase of *Garrulus glandarius*—which is a very efficient acorn disperser that hides seeds underground, especially in areas where seed predation risk is low—might have influenced the emergence of white oak seedlings [104,105].

## 5. Conclusions

The results presented in this paper, though preliminary, show that close-to-nature silviculture intervention—by mimicking natural small-scale gap dynamics—may be safely applied without detrimental impacts on biodiversity in the short-term. The taxonomic distinctness and species diversity indices provide helpful information about biodiversity assessment, and their combined use for monitoring plant community changes and management techniques’ effects on forest ecosystems should be promoted.

The intervention has not appeared to be significantly effective in increasing ornithological biodiversity. However, it appears that changes in the structure of vegetation through silvicultural interventions may have promoted changes at the level of a single bird species. Although our observations were restricted to the first three years following treatment, current dynamics lead us to justify further monitoring activity over time.

The initial response of understorey vegetation is assumed to be a combination of the response to the disturbance of silvicultural operations and changes in the availability of resources, such as light and water [16]. However, it seems that the interventions carried out, and above all the short time lapse of monitoring, were insufficient for more in-depth evaluations of shifts in the vascular plant and bird communities. Nevertheless, our data seem to confirm that governance and management for preserving and favouring ecosystem integrity of old-growth forest patches should focus on dedicated silvicultural treatments aimed at increasing the diversity of plant and bird communities, and hopefully improve their resilience to climate change [4,11,50,106].

We believe that this work can contribute to responding to the request of deepening guidelines and techniques aimed at preserving populations of high compositional and structural value, which both human disturbances and the ongoing climatic change can put at risk of conservation. Although there are numerous references in the scientific literature, this case study concerns a forest community that is peculiar for several reasons, such as the importance of this endemic oak for biodiversity, the geographical position at the southernmost edge of the distribution range of the species, and finally, the application of a long-term forestry project, of which we presented the first steps and outcomes. Moreover, the results obtained contribute to the understanding of Mediterranean old-growth forests, the management of which should be geared towards maintaining biodiversity. Local decision makers and managers of protected areas can benefit from these results to address an adequate management of old-growth forests, also in light of evidence that climate change may interact with forest resilience and management in multifaceted ways.

## Figures and Tables

**Table 1:** Descriptive statistics of the diversity indices computed before and after the application of silvicultural treatment. Results of paired *t*-tests under normal distribution and Wilcoxon signed-rank tests under non-normal distribution.

Index	Time	Selection Thinning								Control							
		Mean	SD	Min	Max	SE	t	df	*p*-Value	Mean	SD	Min	Max	SE	t	df	*p*-Value
*S*	Before	11.57	3.21	8.00	18.00	1.21	−2.97	6	0.025 *	11.50	2.38	9.00	14.00	1.19	−1.97	3	0.144
	After	14.86	3.24	11.00	20.00	1.22				14.75	1.71	13.00	17.00	0.85			
*H′*	Before	1.67	0.41	1.10	2.29	0.16	0.26	6	0.801	1.75	0.28	1.34	1.95	0.14	−0.83	3	0.467
	After	1.65	0.40	1.25	2.44	0.15				1.85	0.15	1.72	2.00	0.08			
*J′*	Before	0.71	0.07	0.59	0.82	0.03	2.59	6	0.041 *	0.71	0.06	0.64	0.77	0.03	1.93	3	0.149
	After	0.64	0.05	0.57	0.71	0.02				0.63	0.03	0.60	0.66	0.01			
1−D	Before	0.72	0.12	0.53	0.85	0.05	0.95	6	0.378	0.76	0.07	0.66	0.82	0.03	−0.66	3	0.558
	After	0.69	0.13	0.52	0.87	0.05				0.78	0.05	0.73	0.83	0.02			
*N*	Before	0.32	0.16	0.12	0.57	0.06			0.272	0.37	0.15	0.23	0.56	0.07	−0.83	3	0.467
	After	0.37	0.25	0.11	0.82	0.09				0.42	0.22	0.19	0.72	0.11			
Δ^+^	Before	82.14	2.60	77.28	84.88	0.98	−1.83	6	0.118	78.90	3.59	74.90	82.73	1.79			0.584
	After	83.29	2.96	76.85	85.92	1.12				79.93	1.45	77.87	81.15	0.73			
*s*Δ^+^	Before	946.01	235.76	651.76	1390.98	89.11	−3.12	6	0.021 *	903.89	169.66	744.55	1052.55	84.83	−2.15	3	0.121
	After	1231.59	234.00	921.80	1537.01	88.45				1179.23	142.11	1049.45	1379.48	71.06			

SD: Standard deviation; SE: Standard error of the mean; t: *t*-test statistic value; df: degrees of freedom; (*) indicates significant differences (*p* < 0.05).

**Table 2:** Bird community and forest specialist species recorded on Mount Egitto. Frequency (number of points with presence/total number of points) and abundance (number of individuals per species per point) of birds detected in the first distance band (0–25 m) before and after treatment. Results of GLMs are also shown.

Scientific Name	Common Name	Forest Specialists	Before		After		GLMs	
			Band _0–25m_		Band _0–25m_			
			Frequency	Abundance	Frequency	Abundance	Thinning	Other Variables
*Aegithalos caudatus*	Long-tailed tit	x			0.10	0.10		
*Buteo buteo*	Common buzzard							
*Linaria cannabina*	Common linnet		0.19	0.24			n.s.	
*Certhia brachydactyla*	Short-toed treecreeper	x	0.29	0.33	0.57	0.67	(+) *	pine plantation (−) *
*Columba palumbus*	Common wood pigeon	x	0.14	0.14	0.10	0.10	n.s.	
*Corvus cornix*	Hooded crow		0.05	0.05				
*Cuculus canorus*	Common cuckoo							
*Cyanistes caeruleus*	Eurasian blue tit	x	0.67	0.76	0.62	0.67	n.s.	
*Dendrocopos major*	Great spotted woodpecker	x	0.14	0.14	0.10	0.10	n.s.	
*Emberiza cia*	Rock bunting				0.05	0.10		
*Emberiza cirlus*	Cirl bunting		0.05	0.05				
*Erithacus rubecula*	European robin	x	0.14	0.14	0.10	0.14	n.s.	pine plantation (+) *
*Fringilla coelebs*	Common chaffinch	x	0.19	0.19	0.29	0.33	(+) *	pine plantation (−) *
*Garrulus glandarius*	Eurasian jay	x	0.05	0.05	0.19	0.24	n.s.	
*Lullula arborea*	Woodlark							
*Oriolus oriolus*	Eurasian golden oriole							
*Parus major*	Great tit	x	0.19	0.19	0.24	0.33	n.s.	
*Periparus ater*	Coal tit	x	0.76	1.19	0.57	0.86	n.s.	
*Phoenicurus ochruros*	Black redstart							
*Phylloscopus collybita*	Common chiffchaff	x	0.57	0.57	0.57	0.57	n.s.	
*Pica pica*	Eurasian magpie							
*Regulus ignicapilla*	Common firecrest	x	0.19	0.19	0.19	0.19	n.s.	
*Sitta europaea*	Eurasian nuthatch	x	0.29	0.48	0.29	0.48	n.s.	pine plantation (−) *
*Sylvia atricapilla*	Eurasian blackcap	x	0.48	0.67	0.33	0.38	n.s.	pine plantation (+) *
*Sylvia cantillans*	Eastern subalpine warbler		0.10	0.19	0.19	0.29	n.s.	
*Troglodytes troglodytes*	Eurasian wren	x	0.29	0.29	0.33	0.33	n.s.	
*Turdus merula*	Common blackbird	x	0.05	0.05	0.05	0.05		
*Turdus viscivorus*	Mistle thrush	x						
*Upupa epops*	Eurasian hoopoe							

Thinning: effect and level of significance of thinning; other variables: effect and level of significance of variables (only if *p* < 0.05); (+) positive effect; (−) negative effect; n.s.: no significant *p*-values; * *p* < 0.05.

**Table 3:** Results of a Wilcoxon signed-rank test under non-normal distribution to check for differences in community indices between the two survey times per count station. Results of GLMs are also shown.

Index	Time	Median	MAD	IQR	*p*-Value	GLMs	
						Thinning	Other Variables
total species richness	Before	5	1.48	2	0.842	n.s.	
	After	5	2.97	3			
total abundance	Before	6	1.48	1	0.791	n.s.	
	After	6	2.97	3			

MAD: median absolute deviation; IQR: interquartile range; thinning: effect and level of significance of thinning; other variables: effect and level of significance of variables (only if *p* < 0.05); n.s.: no significant *p*-values.

**Table 4:** Results of a paired *t*-test under normal distribution to check for differences in forest specialisation community indices between the two survey times per count station. Results of GLMs are also shown.

Index	Time	Mean	SD	SE	t	df	*p*-Value	GLMs	
								Thinning	Other Variables
species richness	Before	4.43	1.86	0.41	−0.795	20	0.436	n.s.	
	After	4.71	1.82	0.40					
abundance	Before	5.38	2.58	0.56	−0.260	20	0.797	n.s.	
	After	5.52	2.23	0.49					
WBCI	Before	6.88	3.08	0.67	−0.260	20	0.797	n.s.	
	After	7.08	2.98	0.65					

SD: Standard deviation; SE: Standard error of the mean; t: *t*-test statistic value; df: degrees of freedom; thinning: effect and level of significance of thinning; other variables: effect and level of significance of variables (only if *p* < 0.05); n.s.: no significant *p*-values.

## Data Availability

The data presented in this study are available in the article and Supplementary Materials.

## Supplementary Materials

The following supporting information can be downloaded at: https://www.mdpi.com/article/10.3390/plants12102053/s1, Table S1: Taxonomy dataset of the vascular plant community recorded in Mount Egitto before and after treatment. We also indicated nemoral species having their optima in the forest environment of the study area; Table S2: Pearson’s correlations between the diversity indices computed before treatment; Table S3: Pearson’s correlations between the diversity indices computed after treatment in the thinning sites; Table S4: Pearson’s correlations between the diversity indices computed in the control sites.
